# Intensified training augments cardiac function, but not blood volume, in male youth elite ice hockey team players

**DOI:** 10.1113/EP091674

**Published:** 2024-07-16

**Authors:** Mads Fischer, Jan S. Jeppesen, Jeppe F. Vigh‐Larsen, Eric J. Stöhr, Magni Mohr, Kate A. Wickham, Lasse Gliemann, Jens Bangsbo, Ylva Hellsten, Morten Hostrup

**Affiliations:** ^1^ The August Krogh Section for Human Physiology, Department of Nutrition, Exercise and Sports (NEXS) University of Copenhagen Copenhagen Denmark; ^2^ Section of Sport Science, Department of Public Health Aarhus University Aarhus Denmark; ^3^ COR‐HELIX (Cardiovascular Regulation and Exercise Laboratory – Integration and Xploration), Institute of Sports Science Leibniz University Hannover Germany; ^4^ Department of Medicine, Division of Cardiology Columbia University Irving Medical Center New York New York USA; ^5^ Department of Sports Science and Clinical Biomechanics, SDU Sport and Health Sciences Cluster (SHSC) University of Southern Denmark Odense Denmark; ^6^ Centre of Health Sciences, Faculty of Health University of the Faroe Islands Tórshavn Faroe Islands

**Keywords:** blood volume, echocardiography, high‐intensity interval training, performance

## Abstract

While it is well‐established that a period of interval training performed at near maximal effort, such as speed endurance training (SET), enhances intense exercise performance in well‐trained individuals, less is known about its effect on cardiac morphology and function as well as blood volume. To investigate this, we subjected 12 Under‐20 Danish national team ice hockey players (age 18 ± 1 years, mean ± SD) to 4 weeks of SET, consisting of 6–10 × 20 s skating bouts at maximal effort interspersed by 2 min of recovery conducted three times weekly. This was followed by 4 weeks of regular training (follow‐up). We assessed resting cardiac function and dimensions using transthoracic echocardiography and quantified total blood volume with the carbon monoxide rebreathing technique at three time points: before SET, after SET and after the follow‐up period. After SET, stroke volume had increased by 10 (2–18) mL (mean (95% CI)), left atrial end‐diastolic volume by 10 (3–17) mL, and circumferential strain improved by 0.9%‐points (1.7–0.1) (all *P *< 0.05). At follow‐up, circumferential strain and left atrial end‐diastolic volume were reverted to baseline levels, while stroke volume remained elevated. Blood volume and morphological parameters for the left ventricle, including mass and end‐diastolic volume, did not change during the study. In conclusion, our findings demonstrate that a brief period of SET elicits beneficial central cardiac adaptations in elite ice hockey players independent of changes in blood volume.

## Abstract (highlights)

- **What is the central question of this study?**
  Can a short 4‐week period of speed endurance training (SET) enhance central cardiovascular function and blood volume in youth elite ice hockey players?
- **What is the main finding and its importance?**
  Only a few weeks of SET enhances several markers of cardiac function in youth elite ice hockey players. These findings emphasize the potential benefits of incorporating SET into the training regimen of elite athletes, contributing valuable insights into optimizing cardiovascular performance in this population.

## 1 INTRODUCTION

The physical demands of professional ice hockey have been surging during the past decades (Cox et al., 1995; Vigh‐Larsen et al., 2019). In contemporary ice hockey, players perform multiple intense actions including rapid changes of direction and fast‐paced skating during brief on‐ice bouts (∼30–60 s) interspersed by a longer duration of passive recovery (∼3–5 min) (Brocherie et al., 2018; Douglas & Kennedy, 2020; Lignell et al., 2018). This requires not only a high anaerobic power and capacity, but also a well‐adapted cardiovascular system and capacity of aerobic energy systems to cope with the continuous demands of an ice hockey match. The average heart rate during ice hockey matches is ∼85% maximum and near‐maximal levels are frequently reached (Lignell et al., 2018; Vigh‐Larsen et al., 2020). Accordingly, training strategies aiming to improve both anaerobic and aerobic exercise capacity in elite ice hockey players are of value.

High‐intensity interval training (HIIT) modalities effectively improve aerobic and anaerobic outcomes but depend on how the training is composed. Speed endurance training (SET), comprising short intervals (10–60 s) at maximum or near‐maximal intensities with long recovery (often 2–6 times the work duration), has proven useful to enhance multiple aspects of intense exercise performance in team sport athletes already accustomed to HIIT (Iaia & Bangsbo, 2010; Mohr et al., 2007; Nyberg et al., 2016). While single bouts of SET mainly target anaerobic energy systems, the repeated bouts during SET elicit a substantial cardiovascular stimulus (Parolin et al., 1999), which is supported by average heart rate of ∼85% of maximum with peaks at >95% of maximum during SET sessions (Gibala et al., 2006; Mohr et al., 2007). Thus, SET appears useful for ice hockey players as it stimulates the relevant energy systems and comprises intervals and recovery periods resembling match‐play scenarios. Whether SET induces cardiac and haematological adaptations in athletes, who are already accustomed to performing a substantial amount of HIIT, remains unknown (Baggish et al., 2008; Sommer Jeppesen et al., 2022).

The heart exhibits a remarkable ability to adapt in response to changes in physical demands, as evidenced by cardiac atrophy caused by bed resting (Saltin et al., 1968; Westby et al., 2016) and eccentric hypertrophy (‘athlete's heart’) caused by high volumes of endurance‐based exercise training (Arbab‐Zadeh et al., 2014; Baggish et al., 2008). However, a gap in the literature exists as to the temporality of cardiac adaptations in elite athletes. Training periods of at least 6–12 weeks have commonly been utilized, as this is the presumed duration required to detect cardiac adaptations in adults (Baggish et al., 2008; Bonne et al., 2014; Hatle et al., 2014). But such regimens are inapplicable to elite ice hockey players who often have periodized training schedules with variations in training intensity and load (intensified training) for a few weeks followed by shorter periods with reductions in training load (Rønnestad et al., 2019). Given that only a few weeks’ HIIT can augment maximal oxygen consumption (${\dot{V}}_{O_{2}\max}$) in elite ice hockey players (Hatle et al., 2014; Rønnestad et al., 2019; Sommer Jeppesen et al., 2022), and that young individuals are more responsive to exercise training (Arbab‐Zadeh et al., 2014; Fujimoto et al., 2010; Levine, 2008; Saltin et al., 1995), cardiac adaptations likely occur more rapidly in young elite ice hockey players than current evidence suggests.

An area of controversy concerning cardiac adaptability is whether early (<12 weeks) training adaptations in cardiac volumes and function relate to blood volume expansion or cardiac remodeling (Bonne et al., 2014; Carrick‐Ranson et al., 2014; Godfrey RJ et al., 2005; Pedlar et al., 2018; Skattebo et al., 2020). In otherwise untrained individuals, there is a temporal association between cardiac and haematological adaptations (Arbab‐Zadeh et al., 2014). It is well described that dehydration and/or blood withdrawal reduces stroke volume and cardiac output (Bonne et al., 2014; González‐Alonso et al., 1995; Lord et al., 2018; Schierbauer et al., 2021; Watanabe et al., 2020). Expansion of intravascular blood volume with a period of exercise training appears to drive cardiac functional and morphological adaptations (Bonne et al., 2014; Perkins et al., 2022; Skattebo et al., 2020). Bonne et al. (2014) attributed blood volume expansion, rather than structural cardiac adaptations, as the main mechanism underlying training‐induced increases in cardiac output, as withdrawal of the blood gained during 6 weeks of HIIT abolished the adaptations. This contrasts with recent findings by Skattebo et al. (2020) who observed that enhancement of peak cardiac output was retained despite removing the blood gained during 10 weeks of endurance training. In that study, adaptation in peak cardiac output was associated with left cardiac ventricular concentric remodeling (Skattebo et al., 2020). However, untrained individuals are not representative of elite athletes, as they present adaptations in almost all cardiac and haematological variables with training (Arbab‐Zadeh et al., 2014; Bonne et al., 2014; Schierbauer et al., 2021). Hence, it is pertinent to examine the temporality of cardiac adaptations incurred from HIIT, as well as their association with changes in intravascular blood volume in elite athletes.

In this study, we investigated the effect of 4 weeks of intensified training, consisting of SET, followed by a period of normalized training (follow‐up), on cardiac morphology and function as well as intravascular blood volume in Under‐20 Danish national team ice hockey players. We hypothesized that 4 weeks of SET would improve cardiac function, while blood volume would remain unaltered.

## 2 METHODS

### 2.1 Participants and ethical approval

From March through June 2021, male youth ice hockey players included in the Danish Under‐20 national team center of the Capital Region were invited to participate in the study. The study was conducted in accordance with the Declaration of Helsinki with no objection from the ethical committee of the Copenhagen Region, Denmark (Journal‐number: 20082260). Written informed consent was provided by all players before participation in the study. For players <18 years of age, their parent or guardian also gave written informed consent. None of the players used prescription medicine, they had no known cardiovascular, metabolic or neurological disease, and they presented with normal echocardiographic assessments. All the tests were carried out at Department of Nutrition, Exercise and Sports (NEXS), University of Copenhagen, Denmark.

### 2.2 Study design

**FIGURE 1:**
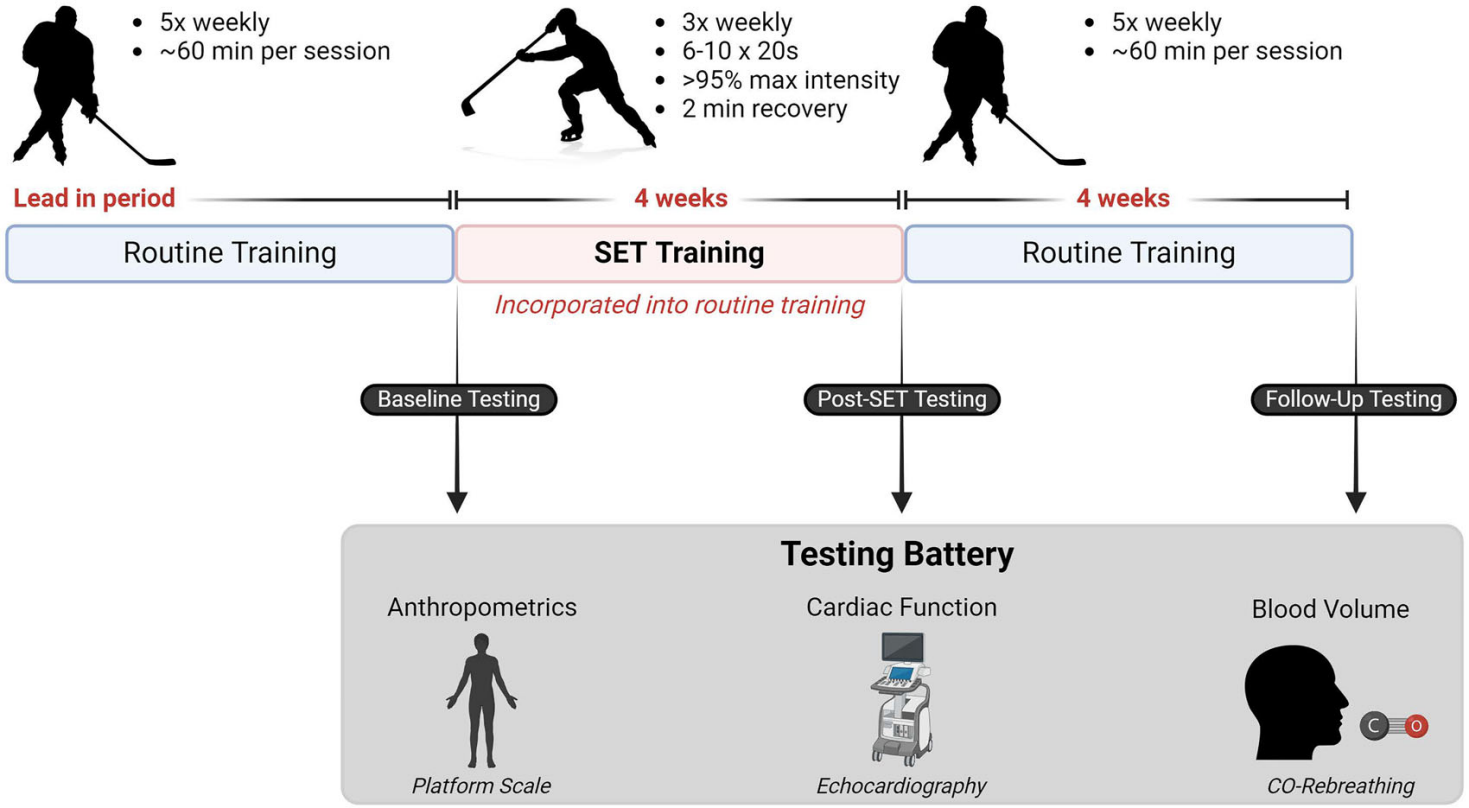
Timeline of the training and the testing battery. SET, speed endurance training; CO, carbon monoxide. Created with BioRender.com.

We utilized a longitudinal study design (Figure 1a) involving a 4‐week training intervention consisting of intensified training with SET replacing parts of the normal training. The SET period was followed by a 4‐week follow‐up period, where the players reverted to their regular weekly training intensity and load. The primary outcome was stroke volume, while the secondary measures were total blood volume, resting heart rate, left ventricle (LV) end‐diastolic volume, and LV systolic function measured as circumferential and longitudinal strain. The study was part of a larger project investigating the utility of SET to improve performance in ice hockey players (Sommer Jeppesen et al., 2022).

### 2.3 Training intervention and follow‐up

Before the training intervention, players trained on ice five times weekly for 60 min per session (Figure 1a). During the SET period, players implemented 6–10 bouts of SET (Week 1: 6 bouts, Week 2: 8 bouts, Weeks 3–4: 10 bouts) as drills performed during the last 15–25 min of the on‐ice training. This consisted of 20 s skating at maximal effort interspersed by 2 min of recovery conducted at three of the up to five weekly training sessions. This period was followed by the 4‐week follow‐up period, in which players engaged in their usual five weekly on‐ice training sessions as they did before the study. The study was conducted ‘off‐season’ during a period with no competitive matches during and two months leading up to the training intervention.

### 2.4 Pre‐ and post‐intervention assessment

Before and after the training intervention and again at follow‐up, players underwent an experimental trial at our laboratory for echocardiographic assessment and intravascular blood volume measurements (Figure 1b). Players were asked to refrain from strenuous physical activity for 24 h before the trial and to refrain from consuming caffeine, nicotine and alcohol for 12 h before. Players arrived at the laboratory and underwent anthropometric measurements of weight and height using a platform scale with an integrated height measuring stick (Kern MPE 250K100PM, Balingen, Germany). After a short rest, a blood sample was collected from the antecubital vein in a 2 mL EDTA tube for analysis of haemoglobin concentration and haematocrit using an automated haematology analyser (Sysmex XN‐450, Sysmex, Kobe, Japan). The players then rested in a supine position for 10 min before transthoracic echocardiography examination in accordance with current echocardiographic guidelines (Lang et al., 2015). Hereafter, blood volume was assessed with the carbon monoxide (CO) rebreathe method (Burge & Skinner, 1995; Siebenmann et al., 2017).

### 2.5 Experimental procedures

#### 2.5.1 Cardiac morphology and function

Transthoracic echocardiography was performed using a GE Vivid E9 ultrasound machine with a 2.5‐MHz transducer (GE Healthcare, Copenhagen, Denmark) according to the current guidelines from the American Society of Echocardiography and the European Association of Cardiovascular Imaging (Lang et al., 2015). All measurements and analyses were conducted during the same time of day by an experienced echocardiographer from ​The Department of Cardiology at Copenhagen University Hospital *Rigshospitalet*. During the examination, participants rested in a supine position and turned on their left side in an airconditioned (23°C) and darkened room. LV peak systolic (*S*′) as well as early (*E*′) and late (*A*′) diastolic mitral annular velocity was measured with pulsed‐wave (PW) tissue Doppler imaging (TDI) averaged from the septal and lateral mitral annulus. The frame rate was set to a minimum of 57 frames per second to ensure the highest possible frame rate while maintaining optimal spatial resolution (Stöhr et al., 2016). Stroke volume was evaluated using the velocity time integral (VTI) method (LVOT × VTI) with the LV outflow tract diameter measured at diastole (LVOT) at each time point (Shahgaldi et al., 2010). LV and atrial volumes were evaluated with Simpson's biplane method (using both two‐chamber and four‐chamber images). To reduce analytical variability, all examinations were analysed offline with EchoPac (software version 203, GE Healthcare), using a minimum of three consecutive cardiac cycles and with automatic tracking and tracing of acoustic markers (LVOT VTI, mitral valve *E*/*A* ratio, mitral annular longitudinal velocities, left atrial volumes, LV volumes). For strain analysis, regions of interest were semi‐automatically traced around the endocardial borders in each (four and two chamber) view, and the software automatically tracked speckles within the region of interest throughout the cardiac cycle, which was done for three cardiac cycles as well. Left atrial strain was assessed using the P‐wave as the QRS reference point and was traced and analysed as detailed by Cameli et al. (2009, 2016). Following analysis with EchoPAC, LV rotation and apical view were assessed using an additional (1.0β14, Stuttgart, Germany) to smooth the raw data with cubic spline interpolation and to generate a total of 1200 data points with 600 data points at the systolic and diastolic cardiac phase, respectively (Stöhr et al., 2015). Due to the acoustic window and thereby poor image quality, only nine players’ LV short axis images were included at baseline, eight after SET and nine after follow‐up.

Prior to the main study, to test for typical error (i.e., test–retest variation) expressed as a coefficient of variation percentage (CV%), the sonographer performed an echocardiographic exam on a separate randomly selected cohort of nine individuals on two consecutive days. *Post hoc* analyses were conducted with the sonographer blinded to the subject information and time to reduce bias. Key measures such as LV end‐diastolic volume by Simpson's biplane, stroke volume by LVOT × VTI, *E*/*e′* ratio, interventricular septum thickness at end‐diastole, left ventricular posterior wall at end‐diastole and heart rate for the nine participants was 6.5%, 4.3%, 3.7% and 7.2%, respectively. Stroke volume by LVOT × VTI was chosen as the main outcome for stroke volume due to the significantly lower CV% and higher accuracy of stroke volume by LVOT × VTI compared to the high CV% of 2D echocardiographic imaging and calculations, which are evident in other studies as well (Billig et al., 2021; Shahgaldi et al., 2010). Supporting information Figure S1 provides a comparison (Bland–Altman analysis) of stroke volume measurements using the LVOT × VTI method and Simpson's biplane methods. Left ventricular mass was calculated using the area–length method with measures collected at end‐diastole as recommended by American Society of Echocardiography and European Association of Cardiovascular Imaging (Lang et al., 2015).

Body surface area (BSA) was calculated according to DuBois and DuBois formula (DuBois & DuBois, 1915):

BSA: $0.202\times {\left(\mathrm{body}\;\mathrm{mass}\right)}^{0.425}\times {\left(\mathrm{height}\right)}^{0.725}$.

### 2.6 Intravascular blood volume

Total intravascular blood volume, plasma volume and haemoglobin mass were measured using a custom‐made rebreathe circuit and calculated using the carbon monoxide rebreathing method as previously described (Burge & Skinner, 1995). Players rested in a supine position with their legs slightly elevated. After 10 min of rest, four capillary blood samples (35 μL in pre‐heparinized glass capillary tubes; Radiometer, Copenhagen, Denmark) were taken from a finger to assess resting carboxyhaemoglobin, whereafter the player was immediately connected to the mouthpiece for the rebreather circuit, which contained a bolus of 1.0 mL/kg 99.997% chemically pure carbon monoxide (CO) (CO N47, Air Liquide, Paris, France) mixed in 5 L 100% oxygen, and rebreathed the gas mixture for 2 min. To verify that no CO was leaking during the 2 min of rebreathing, a portable CO gas analyser (Dräger PAC 6000; Dräger Safety, Lübeck, Germany) with a parts‐per‐million sensitivity to monitor local CO levels was placed nearby. When the 2 min had passed, the player was allowed to breathe freely for 4 min. The portable CO gas analyser was further used to assess end‐tidal CO concentration before and after (4 min after the initiation) the CO‐rebreathing and the remaining amount of CO in the spirometer and breathing bag. Capillary carboxyhaemoglobin was determined from four capillary blood samples using a blood gas analyser (ABL800‐flex, Radiometer) 7 min after the application of CO to allow for adequate circulatory mixing (Siebenmann et al., 2017). Haemoglobin concentration and haematocrit values from the first EDTA blood sample were used for assessment of plasma and red blood cell volumes.

### 2.7 Statistical analysis

SPSS was used for statistical analysis (IBM SPSS Statistics, version 28, IBM Corp., Armonk, NY, USA). Data were tested for normality using the Shapiro–Wilk test and Q–Q plots. Data were normally distributed and are presented as means ± SD. To estimate changes with the SET period and subsequent follow‐up period, we utilized a linear mixed model with time as a fixed effect (baseline vs. SET vs. follow‐up) and subjects as a random effect. Outcome statistics are presented as mean delta changes with 95% confidence intervals and *P*‐values to represent probability. Associations between outcomes were assessed using linear regression (Prism version 9.3.1; GraphPad Software, Boston, MA, USA). α was set at *P* ≤ 0.05. With 11 participants, an α of 0.05, and statistical power (1 − β) of 0.80, this study was adequately powered to detect effect sizes (Cohen's *dz*) of 0.8 or larger.

## 3 RESULTS

### 3.1 Participants

Eleven players volunteered to participate in the study (all Caucasian ethnicity). Upon the initial visit players had an age of 18 ± 1 years, height of 184 cm and body mass of 80.6 ± 11.0 kg (Table 1).

**TABLE 1:** Subjects’ anthropometric characteristics.

	Baseline	*n*	SET	*n*	Follow‐up	*n*
Age (years)	18 ± 1	11		11		11
Height (cm)	184 ± 5	11		11		11
Body mass (kg)	80.6 ± 11.0	11	81.6 ± 10.2	11 (0.10)	81.3 ± 10.2	10 (0.06)
Body surface area (m^2^)	2.03 ± 0.14	11	2.04 ± 0.13	11 (0.08)	2.04 ± 0.14	10 (0.05)

*Note*: Values are expressed as means ± SD. Effect size, measured by Cohen's *dz*, is provided in brackets for changes observed from Baseline to SET, and from SET to Follow‐up. *n* = sample size. Abbreviation: SET, speed endurance training.

### 3.2 Training distribution adherence

Before the intervention, players had a training volume of five weekly on‐ice training sessions of ∼65–70 min duration and two to three weekly full‐body resistance training sessions of ∼60 min duration. Average weekly on‐ice training duration was 356 ± 50 min during the SET period and 332 ± 20 min during the follow‐up period (*P* = 0.489), with 91 ± 7% adherence, as the group completed 120 of 132 individually planned training sessions.

### 3.3 Heart rate and body composition

Body mass did not change significantly during the study (Table 1). Resting heart rate decreased during the SET period (*P* = 0.023) and remained significantly lower (*P* = 0.043) than baseline at the follow‐up period (Table 2). The average heart rate per training session was higher during the SET period than during the follow‐up period (152 ± 1 vs. 148 ± 2 bpm, *P* = 0.009).

**TABLE 2:** Cardiac parameters at baseline and after 4 weeks of speed endurance training (SET) and 4 weeks’ follow‐up.

	Baseline	*n*	SET	*n*	Follow‐up	*n*
**LV structure and volume**						
Mass (g)	190 ± 38	11	198 ± 38	11 (0.06)	199 ± 42	10 (0.04)
Mass index (g/m^2^)	93 ± 15	11	97 ± 15	11 (0.03)	97 ± 17	10 (0.17)
End‐diastolic volume (mL)	159 ± 28	11	160 ± 26	11 (0.05)	159 ± 27	9 (0.03)
End‐diastolic volume index (mL/m^2^)	78 ± 10	11	79 ± 10	11 (0.03)	77 ± 9	9 (0.08)
End‐systolic volume (mL)	72 ± 15	11	70 ± 11	11 (0.11)	73 ± 10	9 (0.16)
End‐systolic volume index (mL/m^2^)	35 ± 6	11	34 ± 5	11 (0.15)	36 ± 4	9 (0.17)
SV_(LVOT×VTI)_ (mL)	87 ± 16.9	11	99 ± 15^a^	11 (0.64)	96 ± 16	9 (0.07)
SV_(LVOT×VTI)_ index (mL/m^2^)	43.4 ± 6.2	11	46.6 ± 7.2^a^	11 (0.52)	46.1 ± 6.0	9 (0.19)
LVOT VTI (cm)	22.3 ± 3.0	11	24.2 ± 3.1^a^	11 (0.54)	24.3 ± 2.8	9 (0.25)
LVOT diameter (mm)	22.3 ± 2	11	22.8 ± 1	11 (0.31)	22.5 ± 1	9 (0.15)
Heart rate (beats/min)	68 ± 11	11	62 ± 9^a^	11 (0.34)	63 ± 10	9 (0.05)
Cardiac output (L/min)	5.9 ± 1.4	11	6.5 ± 1.3	11 (0.46)	6.3 ± 1.0	9 (0.21)
LV systolic function						
Global longitudinal strain (%)	−17 ± 2.0	11	−17.3 ± 1.4	11 (0.20)	−16.6 ± 1.4	9 (0.31)
Global circumferential strain (%)	−18.7 ± 2	9	−19.5 ± 2^a,b^	8 (0.47)	−16.9 ± 3	9 (0.99)
Ejection fraction (%)	55.2 ± 3.5	11	56.1 ± 3.3	11 (0.29)	54 ± 1.8	9 (0.47)
PW TDI *s*′ (cm/s)	10.7 ± 1.3	11	10.2 ± 1.4	11 (0.31)	10.7 ± 1.6	10 (0.36)
Twist peak (deg)	12 ± 5	9	17 ± 6	8 (0.42)	12 ± 4	9 (0.91)
Twist % time to peak (% systole)	56 ± 9	9	46 ± 9	8 (0.62)	46 ± 8	9 (0.05)
Twist velocity peak (deg/s)	85 ± 25	9	103 ± 37	8 (0.18)	96 ± 31	9 (0.36)
LV diastolic function						
Mitral valve *E* velocity (cm/s)	94 ± 12	11	93 ± 14	11 (0.04)	86 ± 10	10 (0.49)
Mitral valve *A* velocity (cm/s)	51 ± 9	11	47 ± 7	11 (0.54)	43 ± 6	10 (0.66)
Mitral valve *E*/*A*	1.9 ± 0.4	11	2.0 ± 0.4	11 (0.43)	2.1 ± 0.4	10 (0.21)
Mitral deceleration time (ms)	224 ± 38	11	191 ± 40^b^	11 (0.79)	215 ± 48	10 (0.65)
PW TDI *e*′ (cm/s)	16.2 ± 1.9	11	16.8 ± 1.2	11 (0.37)	16.5 ± 1.4	10 (0.25)
PW TDI *a*′ (cm/s)	7.4 ± 1.1	11	7.0 ± 1.5	11 (0.31)	6.9 ± 1.5	10 (0.15)
* E*/*e*′	5.8 ± 0.9	11	5.5 ± 0.8	11 (0.31)	5.2 ± 0.4	10 (0.38)
Untwist % time to peak (% diastole)	15 ± 7	9	16 ± 6	8 (0.32)	15 ± 6	9 (0.18)
Untwisting velocity peak (deg/s)	94 ± 38	9	106 ± 31	8 (0.22)	100 ± 29	9 (0.33)
**Right ventricle function and atrial volume**						
TAPSE (cm)	2.8 ± 0.6	11	2.9 ± 0.3	11 (0.09)	2.6 ± 0.4	10 (0.62)
PW TDI *S*′ (cm/s)	15.1 ± 1.9	11	14.9 ± 2.1	11 (0.07)	14.8 ± 2.4	10 (0.06)
PW TDI *E*′ (cm/s)	15.9 ± 3.8	11	16.1 ± 2.0	11 (0.04)	14.7 ± 2.7	10 (0.52)
PW TDI *A*′ (cm/s)	10.3 ± 4.1	11	9.8 ± 2.8^b^	11 (0.11)	8.0 ± 1.5	10 (0.75)
Right atria end‐diastolic volume (mL)	44.2 ± 14.3	10	50.3 ± 14.4	10 (0.43)	45.8 ± 11.2	9 (0.34)
**Left atrial volume and function**						
End‐diastolic volume (mL)	50 ± 14	11	61 ± 15^a^	11 (0.77)	56 ± 14	10 (0.17)
End‐diastolic volume index (mL/m^2^)	24 ± 6	11	30 ± 7^a^	11 (0.81)	28 ± 8	10 (0.06)
End‐systolic volume (mL)	20 ± 9	11	29 ± 8^a^	11 (0.90)	25 ± 7	10 (0.25)
End‐systolic volume index (mL/m^2^)	10 ± 4	11	14 ± 4^a^	11 (0.91)	13 ± 4	10 (0.17)
Ejection fraction (%)	61 ± 8	11	54 ± 6^a^	11 (0.94)	55 ± 7	10 (0.24)
Contractile strain (%)	11.5 ± 2.6	11	10.9 ± 1.4	11 (0.28)	10.9 ± 2.1	10 (0.05)
Conduit strain (%)	34.9 ± 4.9	11	34.5 ± 4.7	11 (0.28)	33.8 ± 4.6	10 (0.26)
Reservoir strain (%)	46.4 ± 7.3	11	45.4 ± 5.8	11 (0.29)	44.8 ± 6.3	10 (0.21)

*Note*: Values are expressed as means ± SD. Effect size, measured by Cohen's *dz*, are provided in brackets for changes observed from Baseline to SET, and from SET to Follow‐up. ^a^SET different from baseline, *P *< 0.05. ^b^SET different from follow‐up, *P *< 0.05. Abbreviations: LA EDV, left atria end‐diastolic volume; PW TDI—*s*′, *e*′, *a*′, systolic myocardial, early diastolic myocardial relaxation and atrial contraction velocity by pulsed wave tissue Doppler imaging; SET, speed endurance training; TAPSE, right ventricular function by tricuspid annular plane systolic excursion; VTI, velocity time integral derived stroke volume.

### 3.4 Cardiac dimensions

LV mass indexed to BSA, end‐diastolic volume, systolic volume and ejection fraction did not change significantly during the SET period or subsequent follow‐up period (Table 2). While stroke volume by LVOT × VTI increased by 10% (2–18 mL, *P* = 0.025) during the SET period and was maintained during the follow‐up period, no significant changes were evident when using Simpson's biplane method (−4 to 10 mL, *P* = 0.436). The percentage difference between methods was 1.9 ± 10.2, 8.9 ± 11.7 and 10.8 ± 10.8 % at baseline, SET and follow‐up, respectively. There was a high goodness of fit between the stroke volume assessed with the two methods (*R* ^2^: baseline, 0.64; SET, 0.61; Follow‐up, 0.73) and significant correlation at all measuring times (baseline *P* < 0.001; SET *P* = 0.005; Follow‐up *P* = 0.007).

### 3.5 LV systolic function

Global LV circumferential strain increased with SET (−0.01 to −1.17%‐points, *P* = 0.030) and returned to baseline levels during the follow‐up period (Table 2). LV ejection fraction, global LV longitudinal strain, and twist parameters (peak twist, time to peak, and twist velocity) did not change during the SET period and follow‐up period (Table 2).

### 3.6 LV diastolic function

Average values of septal and lateral, early (*e*′) and active (*a*′) mitral annular longitudinal velocities and diastolic twist parameters (untwist time to peak and untwist velocity) did not change throughout the study, nor did early (*E*) and active (*A*) mitral valve inflow velocities, and hence *E*/*A* ratio (Table 2). However, mitral valve *E* deceleration time decreased non‐significantly (*P* = 0.069) during the SET period and increased significantly (*P* = 0.044) back to baseline levels during the follow‐up period (Table 2).

### 3.7 Right ventricular function

No significant changes were observed in right ventricular (RV) function as measured by tricuspid annular plane systolic excursion (TAPSE) and tricuspid annular plane PW TDI *S*′ or *E*′ during the study (Table 2).

### 3.8 Left atrial function and dimensions

Left atrial end‐diastolic volume and end‐systolic volume increased 23% (*P* = 0.007) and 42% (*P* = 0.001) during the SET period, respectively, and remained higher than baseline at follow‐up (Table 2). Compared to baseline, left atrial ejection fraction was lower after the SET period (*P* = 0.013) and was reverted to baseline levels at follow‐up (Table 2). Left atrial contractile, conduit and reservoir strain did not change significantly during the study (Table 2).

### 3.9 Intravascular blood volume and haematological parameters

No apparent changes were observed in total blood volume, plasma volume, red cell volume or haemoglobin mass during the SET period or subsequent follow‐up period (Table 3).

**TABLE 3:** Haematological characteristics.

	Baseline	*n*	SET	*n*	Follow‐up	*n*
Total blood volume (L)	6.31 ± 0.67	11	6.37 ± 0.82	11 (0.08)	6.22 ± 0.77	11 (0.19)
Plasma volume (L)	3.5 ± 0.3	11	3.5 ± 0.5	11 (0.06)	3.4 ± 0.4	11 (0.25)
Red cell volume (L)	2.8 ± 0.4	11	2.8 ± 0.4	11 (0.08)	2.8 ± 0.4	11 (0.10)
Haemoglobin mass (g)	907 ± 123	11	926 ± 119	11 (0.16)	915 ± 120	11 (0.09)
Haemoglobin mass per body mass (g/kg)	11.3 ± 0.7	11	11.4 ± 0.8	11 (0.13)	11.1 ± 0.5	11 (0.49)
Haematocrit (%)	44.3 ± 2.2	11	44.1 ± 2.0	11 (0.12)	44.7 ± 1.4	11 (0.25)
Haemoglobin (g/dL)	14.5 ± 0.63	11	14.40 ± 0.57	11 (0.37)	14.72 ± 0.61	11 (0.28)

*Note*: Values are expressed as means ± SD. Effect size, measured by Cohen's *dz*, are provided in brackets for changes observed from Baseline to SET, and from SET to Follow‐up. Abbreviation: SET, speed endurance training.

### 3.10 Individual relationship between cardiac function, volumes and haemodynamics

**FIGURE 2:**
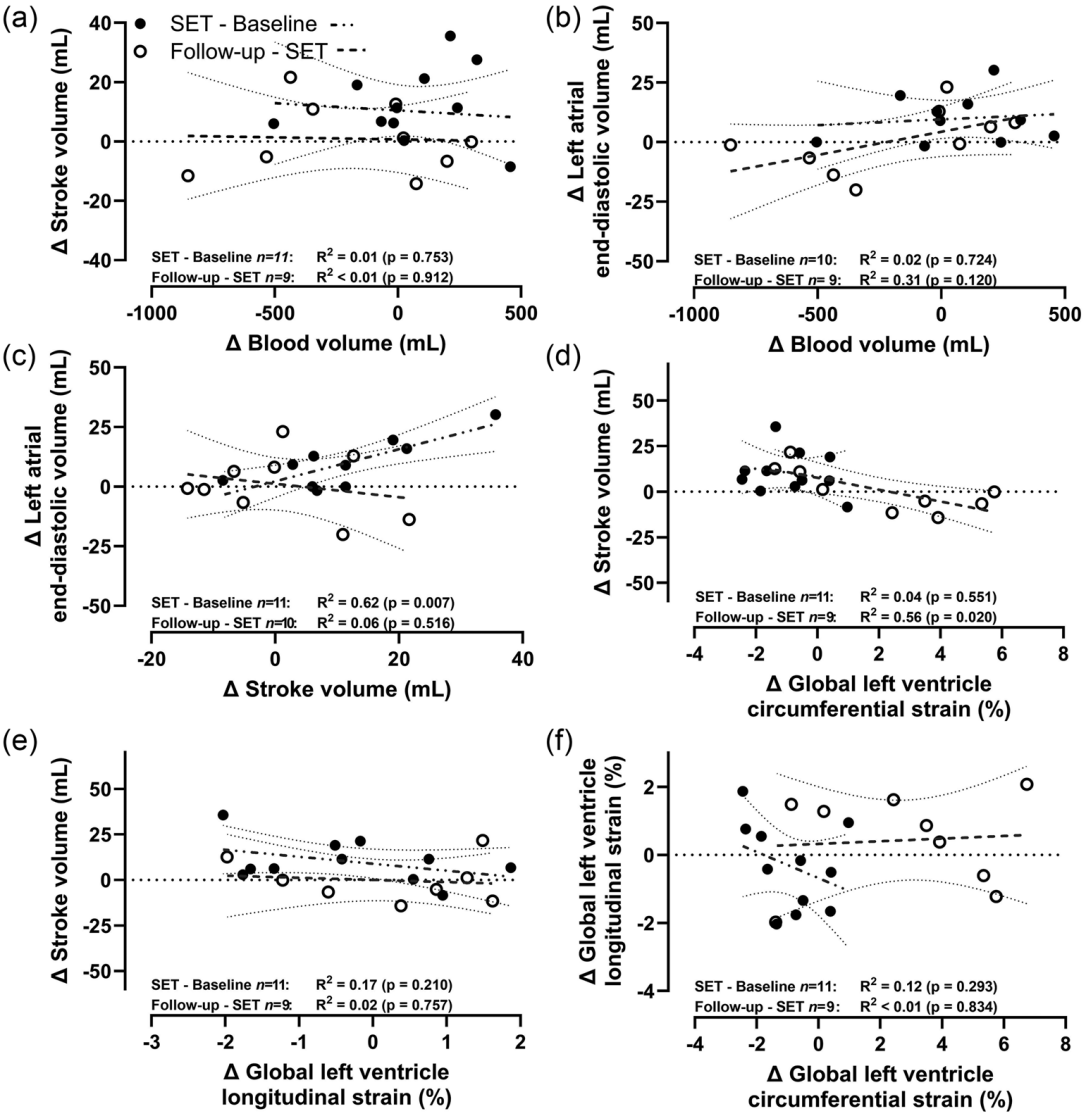
Linear regression for association between changes (delta) in key parameters: stroke volume, blood volume, left atrial end‐diastolic volume, global left ventricular circumferential strain, and global left ventricular longitudinal strain. Filled circles show changes from baseline to SET. Open circles show changes from SET to follow‐up. Regression lines shown the trend of all deltas (filled and open circles) with their 95% confidence interval. SET, speed endurance training.

Change in intravascular blood volume from baseline did not correlate with change in stroke volume during the SET period (*R* ^2^ = 0.01, *P* = 0.753) or from the SET period to the subsequent follow‐up period (*R* ^2^ < 0.01, *P* = 0.912) (Figure 2a). In addition, while change in LA end‐diastolic volume during the SET period did not correlate with change in intravascular blood volume from baseline or from the SET period to follow‐up, it correlated slightly when combining changes across all periods (*R* ^2^ = 0.22, *P* = 0.041) (Figure 2b).

Change in stroke volume during the SET period from baseline did not correlate with change in global LV longitudinal strain (*R* ^2^ = 0.17, *P *< 0.210) (Figure 2e) or global LV circumferential strain (*R* ^2^ = 0.04, *P* = 0.551) (Figure 2d), but did correlate with LA end‐diastolic volume (*R* ^2^ = 0.62, *P* = 0.007) (Figure 2c). Change in stroke volume from after the SET period to the subsequent follow‐up period correlated with change in global LV circumferential strain (*R* ^2^ = 0.56, *P* = 0.004) (Figure 2d) but not with change in LA end‐diastolic volume (*R* ^2^ = 0.06, *P* = 0.516) (Figure 2e) or global LV longitudinal strain (*R* ^2^ = 0.02, *P* = 757) (Figure 2c). Change in global LV longitudinal strain did at no time correlate with the change in global LV circumferential strain (Figure 2f).

## 4 DISCUSSION

The key finding of this study was that 4 weeks of SET improved markers of left cardiac function, including LV global circumferential strain and stroke volume as well as lowered resting heart rate, in youth national team ice hockey players. These adaptations occurred without changes in intravascular blood volume.

**FIGURE 3:**
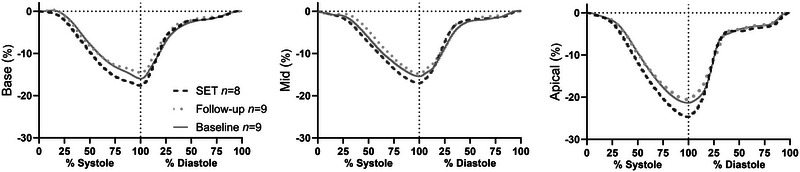
Left ventricular circumferential strain acquired by parasternal short‐axis images at the level of the mitral valve (Base), papillary muscle (Mid), and at the apex (Apical) at baseline (continuous line), in response to SET (dashed line) and at follow‐up (dotted line). SET, speed endurance training.

The 10% increase in resting stroke volume in the current young athlete cohort with 4 weeks of SET is of a similar magnitude to that observed with 6–12 weeks of endurance exercise in studies evaluating cardiac function in non‐athletic younger populations (Arbab‐Zadeh et al., 2014; Bonne et al., 2014; Hatle et al., 2014). We also found SET to elicit beneficial adaptations in other markers of LV function, including greater LV circumferential strain and left atrial end‐diastolic volume, as well as lowered resting heart rate (Table 2, Figure 3). The greater LV circumferential strain indicates that SET predominantly improved systolic function, as circumferential strain represents the change in distance between myocardial segments during systole (Lang et al., 2015). Such adaptations could facilitate stroke volume during exercise and explain the reduced (−12 bpm) heart rate observed during submaximal cycling at 150 W after SET as previously shown in this cohort (Sommer Jeppesen et al., 2022). While the current findings do not include cardiac function measured during stress, fixed‐load cycling revealed a reduction in heart rate for the same intensity indirectly indicates an increased stroke volume. Additionally, the observed rise in ${\overset{\cdot }{V}}_{O_{2}\max}$ with similar heart rates likewise would imply such adaptation. Hence, interval training at near‐maximal intensities can induce rapid improvements in cardiac function even in highly trained individuals. Although it may be argued that the Danish players were tier 4 class athletes (McKay et al., 2022) and not world‐class athletes (Vigh‐Larsen et al., 2019), they displayed similar cardiac characteristics as 18–19‐year‐old pre‐draft‐eligible (combine) hockey players invited to the National Hockey League (NHL) (Ong et al., 2017), supporting the notion that the findings apply to elite hockey players.

A noteworthy finding was that SET induced an apparent disproportionate orientation and regionalization of the adaptation in LV function, as LV longitudinal strain did not adapt concomitantly with circumferential strain (Figure 3). This could potentially be a consequence of the exercise intensity elicited during sessions of SET, imposing greater contributions from circumferential than longitudinal strain to stroke volume (Doucende et al., 2010). Indeed, longitudinal strain plateaus when the exercise intensity reaches that corresponding to 20% maximal aerobic power, which is well below the intensity elicited during bouts of SET (Doucende et al., 2010). The disproportionate orientation of adaptation observed in LV strain (longitudinal vs. circumferential) underpins the importance of examining short‐axis parameters when performing echocardiographic measurements, as adaptations in longitudinal strain can underestimate apical adaptations and may be less responsive to high‐intensity training.

Despite SET effectively enhancing parameters of cardiac function, we observed no parallel changes in intravascular blood volume—neither during the SET period nor during the subsequent follow‐up period. This contrasts the findings by Bonne et al. (2014) of a 7% increase in blood volume with 6 weeks of high‐intensity interval training that also predicted much of the increase in cardiac output. However, the participants in the study by Bonne et al. (2014) were untrained with a blood volume of around 5.7 L, which likely explains the pronounced blood volume expansion (+7%) induced by the training as compared to the youth athletes in the present study with blood volumes of 6.3 L before SET. The observation that the elite youth ice hockey players investigated herein improved multiple markers of cardiac function despite no concomitant changes in intravascular blood volume challenges blood volume as a key factor driving cardiac functional changes in youth athletes exposed to shorter periods of intensified training. Rather, our findings suggest that the enhanced systolic function achieved with SET seems to be the main driver of exercise‐induced improvements in stroke volume. This agrees with the findings by Skattebo et al. who observed that cardiac functional adaptations to a 10‐week period of endurance training were retained when controlling for blood volume in untrained individuals (Skattebo et al., 2020).

Changes in left atrial size and strain are often used as markers of changes in intravascular pressure and blood volume (Cameli et al., 2016; Parsons et al., 2020). While this may be a useful day‐to‐day assessment, as acute changes in blood volume affect cardiac chamber size (Arbab‐Zadeh et al., 2014; Bonne et al., 2014; Lord et al., 2018; Parsons et al., 2020), their relation over longer periods (i.e. several months) is less clear (Arbab‐Zadeh et al., 2014; Bonne et al., 2014; Carrick‐Ranson et al., 2014). While ejection fraction of the left ventricle remained unaffected, a significant reduction was observed in left atrial ejection fraction (EF%), despite the maintenance of left atrial stroke volume. The reduced left atrial EF% could be related to a compensatory response to the elevated preload, as indicated by the increased left atrial size and LV stroke volume (Elliott et al., 2023; Parsons et al., 2020). In situations where LV preload is increased, the left atrium in healthy individuals responds by accommodating a greater volume of blood (Elliott et al., 2023), which could potentially lead to a reduction in its EF%. This reduced EF% may serve as an adaptive mechanism, allowing for a greater functional reserve and facilitating improved ventricular filling during increased exercise stress (Elliott et al., 2023; Esfandiari et al., 2019). While other studies include atrial end‐diastolic volume, they often omit end‐systolic volumes (D'Ascenzi et al., 2012; Król et al., 2016), thereby limiting the literature on atrial ejection fraction in the healthy individuals and athletes.

We found no apparent temporal association between changes in stroke volume and blood volume induced by SET nor during the subsequent normalization of training (Table 2, Figure 2b,c). This suggests that left atrial size and strain should not be used to estimate intravascular blood volume in young athletes.

In summary, our findings demonstrate that only 4 weeks of SET improves several measures of cardiac function, including stroke volume, global LV circumferential strain, and left atrial end‐diastolic volume in youth elite athletes. The fact that several of these adaptations reverted to baseline levels after only a few weeks of return to normal training load underscores the rapid cardiac adaptability in youth athletes and sensitivity to alterations in training intensity. The temporal cardiac changes occurred independently of changes in intravascular blood volume, which suggests that blood volume changes are not the cause of change in LV systolic function in response to a short period of intensified training and subsequent normalization of training load in youth athletes.

### 4.1 Considerations

We acknowledge that the small sample size associated with the strict inclusion criteria of youth national team level players, along with the variability of the measures, may have resulted in some observations in the current study being underpowered. However, for measures such as haemoglobin mass, studies with similar durations have found much greater effects (approximately 10% increases) in untrained individuals (Montero et al., 2017). In contrast, studies involving trained subjects, similar to those in our study, show inconsistent results (Helgerud et al., 2007; Montero & Lundby, 2017).

While the subjects in the current study were part of a larger randomized crossover‐designed study investigating the effectiveness of SET to improve performance in ice hockey players, the participants in the current study all underwent the same period of training and follow‐up, as they were from the same region and were therefore not randomized.

We chose LVOT x VTI method for the main outcome of stroke volume as pilot testing from our lab and a literature review indicated a notably higher accuracy as compared to Simpson's biplane method (Parsons et al., 2020; Shahgaldi et al., 2010). The choice was further substantiated by the lower heart rates observed in our study both during rest and during fixed load cycling (150 W) (Sommer Jeppesen et al., 2022), which supports the LVOT × VTI method's reliability.

A limitation of our study was the absence of female athletes. This was due to the specific cohort we were able to include, and we recognize that extrapolating these findings to female athletes would not be scientifically justified given that sex significantly affects cardiac morphological and electrical remodelling to exercise training (D'Ascenzi et al., 2020; Howden et al., 2015). Further research is needed to elucidate how short‐term exercise affects the cardiac function and morphology in female athletes.

## AUTHOR CONTRIBUTIONS

Morten Hostrup, Jeppe F. Vigh‐Larsen, Jan S. Jeppesen, Magni Mohr, and Jens Bangsbo designed the study. Mads Fischer, Jan S. Jeppesen, and Kate A. Wickham contributed to the collection of data. Morten Hostrup and Jens Bangsbo conceptualized the study. Mads Fischer, Morten Hostrup, and Eric J. Stöhr performed data analysis and interpretation. Mads Fischer and Morten Hostrup drafted the manuscript. All authors critically revised the manuscript. All authors have read and approved the final version of this manuscript and agree to be accountable for all aspects of the work in ensuring that questions related to the accuracy or integrity of any part of the work are appropriately investigated and resolved. All persons designated as authors qualify for authorship, and all those who qualify for authorship are listed.

## CONFLICT OF INTEREST

None.

## Supporting information

Supplemental Figure 1. Bland‐Altman analysis that compares stroke volume measurements obtained through Simpson's biplane method with those derived from the left ventricular outflow tract (LVOT) velocity‐time integral (VTI) method.

## Data Availability

The datasets generated during and/or analysed during the current study are available from the corresponding author on reasonable request.
