# Supplementary material for: Intensified training augments cardiac function, but not blood volume, in male youth elite ice hockey team players
Source: Exp Physiol. 2024 Jul 16;110(5):755–66. doi: 10.1113/EP091674 (PMC12053869; doi:10.1113/EP091674)
Supplement: Supplementary file 1 — Supplemental Figure 1. Bland‐Altman analysis that compares stroke volume measurements obtained through Simpson's biplane method with those derived from the left ventricular outflow tract (LVOT) velocity‐time integral (VTI) method. [file EPH-110-755-s001.docx]

Supplemental Figure 1: Bland-Altman analysis that compares stroke volume measurements obtained through Simpson’s biplane method with those derived from the left ventricular outflow tract (LVOT) velocity-time integral (VTI) method.
